# Multiple Endocrine Neoplasia Type 1 (MEN1) Presenting as an Invasive Macroprolactinoma Complicated by Acute Bacterial Meningitis

**DOI:** 10.7759/cureus.20086

**Published:** 2021-12-01

**Authors:** Sindhura Bandaru, Amruta Jaju, Sukesh Manthri, Chaitanya Mamillapalli, Michael G Jakoby

**Affiliations:** 1 Endocrinology, Terrebonne General Medical Center, Springfield, USA; 2 Endocrinology, Southern Illinois University School of Medicine, Springfield, USA; 3 Oncology, Mary Bird Perkins Cancer Center, Houma, USA; 4 Endocrinology, Springfield Clinic/Southern Illinois University School of Medicine, Springfield, USA

**Keywords:** csf rhinorrhea, male predisposition, bacterial meningitis, macroprolactinoma, multiple endocrine neoplasia type 1 (men1)

## Abstract

Multiple endocrine neoplasia type 1 (MEN1) is a rare autosomal dominant disorder characterized by tumors of parathyroid, anterior pituitary, and pancreatic islet cells. Pituitary adenomas in MEN1 can be aggressive and invade surrounding structures including the skull base. However, acute bacterial meningitis in patients with newly diagnosed macroprolactinomas is an exceptional finding. We present the case of a young man with suppurative meningitis complicating an invasive macroprolactinoma as the initial manifestation of MEN1.

A 33-year-old male was admitted to the hospital with fever, headache, and nuchal rigidity and subsequently diagnosed with *Haemophilus influenzae* bacterial meningitis. Computed tomography (CT) and subsequent magnetic resonance imaging (MRI) of the sella turcica revealed a 5 x 3.5 cm pituitary mass invading both cavernous sinuses and the left sphenoid sinus. Laboratory evaluation was notable for significantly elevated serum prolactin level (2,484 ng/mL, 2.6-13.2) and evidence of hypopituitarism. Primary hyperparathyroidism was indicated by hypercalcemia (13.5 mg/dL, 8.5-10.5), low serum phosphorus (2.0 mg/dL, 2.5-4.9), and elevated intact parathyroid hormone (PTH) level (290 pg/mL, 15-60). No visual field deficits were identified. The patient was managed with hydrocortisone, levothyroxine, and cabergoline. However, cerebral spinal fluid (CSF) rhinorrhea compelled subtotal transsphenoidal resection of the tumor and repair of the CSF leak. Three-and-a-half gland parathyroid resection was performed after recovery from pituitary surgery and successfully treated hypercalcemia. Abdominal MRI revealed a 1.2 cm cystic mass in the neck of the pancreas, and pancreatic polypeptide was approximately fourfold elevated. A clinical diagnosis of MEN1 was made based on the occurrence of macroprolactinoma, multiple parathyroid adenomas, and pancreatic findings. This case appears to be the first in which bacterial meningitis was the initial presentation of an invasive macroprolactinoma in a patient with MEN1.

## Introduction

Multiple endocrine neoplasia type 1 (MEN1) is a rare autosomal dominant disorder caused by mutations in the gene MEN1 that maps to chromosome 11q13 and codes for the nuclear protein menin [1]. MEN1 mutations are distributed across the entire coding region of the gene, and most are truncating mutations that are predicted to disrupt interactions between menin and other proteins important in cell cycle regulation and proliferation [1]. MEN1 manifests primarily as tumors of the parathyroid glands, anterior pituitary, and endocrine pancreas [2]. Additional tumors, including duodenal gastrinomas, adrenocortical adenomas, bronchial and thymic carcinoid tumors, lipomas, angiofibromas, and collagenomas also occur more frequently in MEN1 than in the general population [2]. The diagnosis of MEN1 is made clinically by the occurrence of two or more primary tumors or the occurrence of at least one MEN1-associated tumor in the family member of a known MEN1 patient [2].

Pituitary tumors are recognized as the initial manifestation of MEN1 in 15%-20% of cases, and most pituitary tumors in MEN1 are macroprolactinomas [3]. Macroprolactinomas in MEN1 are more likely to be locally invasive than sporadic macroprolactinomas, and they may be less responsive to dopamine agonists than sporadic tumors [3]. We present a case of MEN1 presenting as bacterial meningitis due to a macroprolactinoma invading an adjacent sphenoid sinus. This case was presented at ENDO 2015 (97^th^ Annual Meeting of the Endocrine Society) on March 5-8, 2015.

## Case presentation

**Figure 1:**
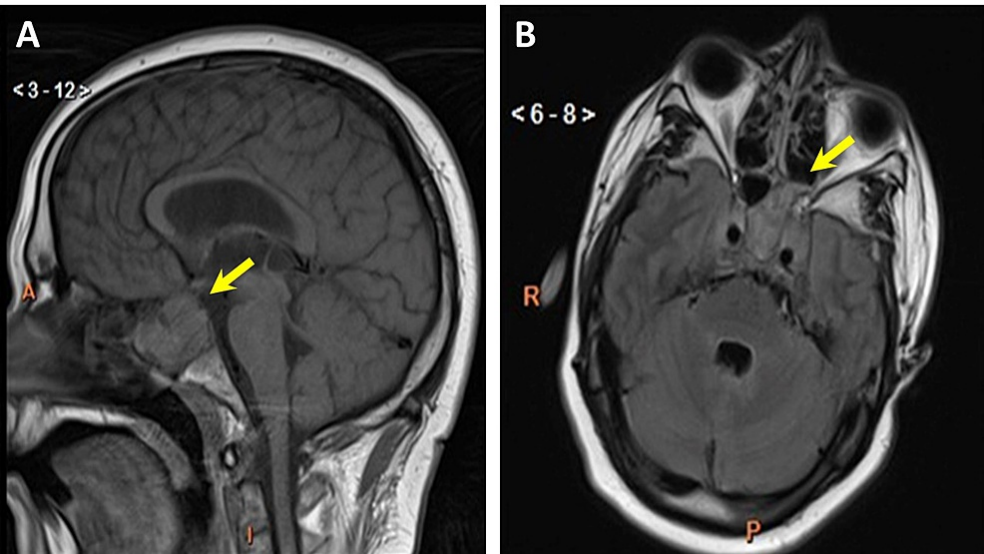
MRI with sella protocol Sagittal (A) and axial (B) magnetic resonance images of a large pituitary mass (yellow arrows) with extension into the left sphenoid sinus (B).

A 33-year-old male presented to the emergency department with fever, chills, sore throat, headache, and stiff neck. The temperature was elevated at 38.5 ^o^C, and the patient was both tachycardic (105 bpm) and tachypneic (respiratory rate 38/min). He was alert but disoriented, though no focal neurological deficits were noted. Computed tomography (CT) of the head revealed a 5 x 3.5 cm sellar mass extending into the cavernous sinuses and left sphenoid sinus, and initial imaging findings were later confirmed by magnetic resonance imaging (MRI, Figures 1A, 1B). Lumbar puncture yielded cloudy cerebral spinal fluid (CSF) with high leukocyte count (27,405/mm^3^, 0-5) and proportion of neutrophils (88%). CSF cultures grew *Haemophilus influenzae*, and the patient fortunately experienced a full recovery after two weeks of treatment with ceftriaxone.

**Figure 2:**
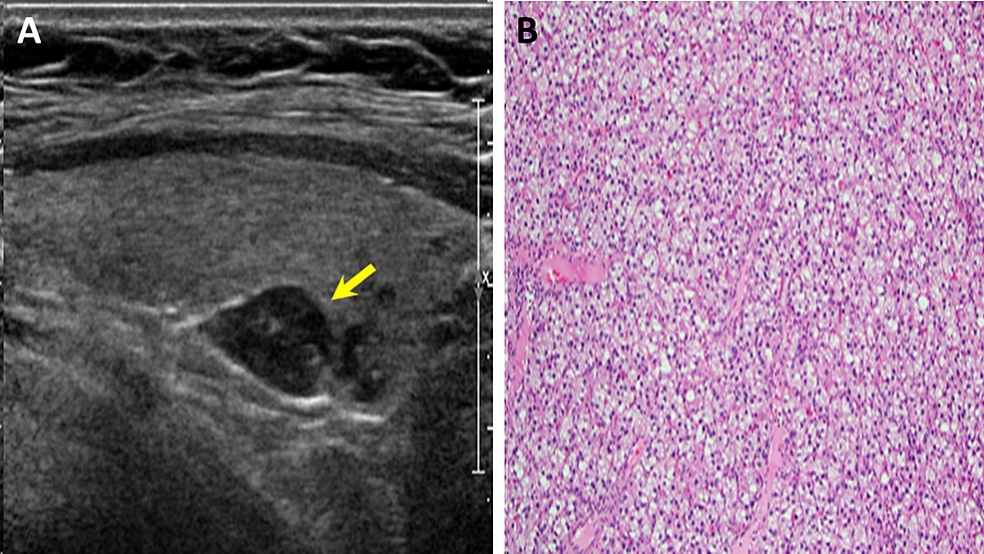
Parathyroid adenoma (A) Neck ultrasound demonstrating an enlarged (1.5 x 0.7 x 1.0 cm) right inferior parathyroid gland (yellow arrow). (B) Hematoxylin and eosin stain of parathyroid adenoma showing a hypercellular, homogenous tumor composed of chief cells and scattered oxyphil cells.

**Figure 3:**
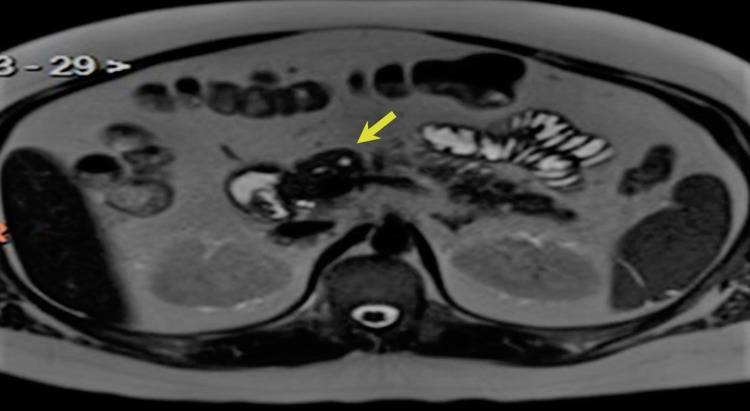
Pancreatic neuroendocrine tumor Abdominal MRI demonstrated a 1.2-cm cystic mass in the neck of the pancreas (yellow arrow).

Serum prolactin level on admission was significantly elevated (2,484 ng/mL, 2.6-13.2), confirming a diagnosis of macroprolactinoma. Evaluation of anterior pituitary function indicated panhypopituitarism. Hypercalcemia, low serum phosphorus, and unequivocally elevated parathyroid hormone (PTH) level indicated primary hyperparathyroidism (PHPT). Relevant endocrine laboratory results are presented in Table 1. Parathyroid scintigraphy showed increased radiotracer uptake in all four parathyroid glands, and ultrasonography demonstrated parathyroid gland enlargement (Figures 2A, 2B). Though there was no family history, the concurrence of a macroprolactinoma and PHPT permitted a clinical diagnosis of MEN1 and prompted evaluation for pancreatic masses. An abdominal MRI was notable for a 1.2 cm cystic mass in the neck of the pancreas (Figure 3), and several additional subcentimeter masses scattered throughout the body and tail of the pancreas were visualized by endoscopic ultrasonography. Pancreatic polypeptide (PP) was subsequently found to be elevated (Table 1).

**Table 1:** Key endocrine laboratory results *Measured from blood drawn at approximately 8 AM.

Parameter	Result	Reference range/expected
Prolactin (ng/mL)	2,484	2.6-13.2
Calcium (mg/dL)	13.5	8.5-10.4
Phosphorus (mg/dL)	2.0	2.5-4.6
Intact PTH (pg/mL)	290	12-88
Pancreatic polypeptide (pg/mL)	1,610	0-435
*Morning cortisol (mg/dL)	1.6	≥ 15
*Morning ACTH (pg/mL)	7	7-69
*Morning TSH (mIU/L)	0.28	0.34-5.60
*Morning free T3 (pg/mL)	2.2	2.5-3.9
*Morning testosterone (ng/mL)	67	270-1070
*Morning LH (mIU/mL)	3.1	1.2-8.6

**Figure 4:**
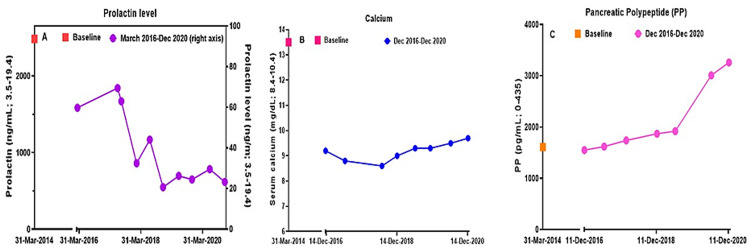
Time courses for the patient's MEN 1 manifestation (A)  Serum prolactin levels after pituitary surgery and during four years of treatment with cabergoline 0.5 mg 4 d/wk. (B) Serum calcium levels following 3.5 gland parathyroidectomy. (C) Pancreatic polypeptide level at initial presentation and over four years of surveillance.

Hydrocortisone, thyroxine, and testosterone were started for management of hypopituitarism, and cabergoline was also started for treatment of hyperprolactinemia. Approximately four weeks later, cerebrospinal fluid (CSF) rhinorrhea occurred, and the patient subsequently underwent transsphenoidal subtotal resection of his macroprolactinoma and repair of the CSF leak. Cabergoline was then restarted, and prolactin levels following surgery and dopamine agonist treatment improved to consistently < 50 ng/mL. A three-and-a-half parathyroid gland resection was performed, resulting in the resolution of hypercalcemia. Annual imaging and serological surveillance have not shown any progression of the patient’s pancreatic masses. The patient’s long-term metabolic course is summarized in Figures 4A-4C.

## Discussion

Key characteristics of macroprolactinomas complicated bacterial meningitis before medical or surgical management, including this case, are presented in Table 2. Though MEN1-associated prolactinomas tend to be larger and are more likely to be locally invasive than sporadic prolactinomas [3], this appears to be the first reported case of MEN1 diagnosed with a macroprolactinoma extending outside the sella turcica and presenting as bacterial meningitis.

**Table 2:** Case reports of macroprolactinomas presenting with bacterial meningitis prior to surgery or medical management. ^†^Ectopic macroprolactinoma originating in the right sphenoid sinus; NR – not reported

Case	Age (y)	Gender	Size (cm)	CSF rhinorrhea	Organism	MEN1
Current case	33	Male	5.0	No	H. influenzae	Yes
Bilo 1984 [4]	42	Male	NR	Yes	NR	No
Onoda 1992 [5]	44	Male	3 cm	No	Group A Streptococcus	No
Utsuki 2004 [6]	69	Male	NR	No	S. pneumoniae	No
Honegger 2009 [7]	64	Male	NR	No	S. pneumoniae	No
Robert 2010 [8]	32	Female	NR	No	S. pneumoniae	No
Chentli 2013 [9]	22	Male	5.8	No	NR	No
Chentli 2013 [9]	49	Male	4.7	Yes	NR	No
Chentli 2013 [9]	25	Male	3.0	No	S. pneumoniae	No
Boscoli 2014 [10]	31	Male	NR	No	NR	No
Margari 2014 [11]	56	Male	NR	No	S. pneumoniae	No
Aslan 2014 [12]	50	Male	NR	Yes	S. oralis	No
Bouchal 2015 [13]	48	Female	3.0	Yes	S. mitis	No
Akinduro 2018 [14]	48	Female	2.0^†^	Yes	S. pneumoniae	No

Despite a strong female predominance for both sporadic [15] and MEN1-associated prolactinomas [3], the significant majority (11/14, 79%) of macroprolactinomas with bacterial meningitis on initial presentation have occurred in men. Though a tendency to larger prolactinomas in men than women has been attributed to delayed evaluation, sex-related differences in growth as indicated by higher Ki-67 immunoreactivity and dopamine agonist resistance also appear to predispose men to macroprolactinomas [16]. In a recent series of giant prolactinomas (≥ 4 cm in largest dimension), the significant majority of patients (63/71, 89%) were men [17]. This patient qualified for a diagnosis of giant prolactinoma, though the size of prolactinomas presenting initially with bacterial meningitis is reported in too few cases to draw conclusions regarding tumor size and risk of infection.

*Streptococcus pneumoniae* is the second most frequent cause of bacterial pneumonia in the United States (USA) after *Neisseria*​​​​​​​* meningitidis* [18], and it was the most frequently identified etiology of meningitis among cases of macroprolactinomas presenting initially with suppurative meningitis for which the bacteriology was reported (6/10, 60%). Three other cases [5,12,13] were due to Streptococcal species known to be present in the oropharynx and nasopharynx. This patient is apparently the first with bacterial meningitis as the initial presentation of prolactinoma for which *H. influenzae* was the causal organism. He was born in the USA in 1980, seven years before the current *Haemophilus* influenza type b (Hib) vaccine was licensed in the USA. The patient was generally healthy, and he had no obvious clinical factors predisposing to *H. influenzae* infection such as chronic lung disease, immunodeficiency, tobacco use, or excessive alcohol consumption. It remains unclear what factors predisposed our patient to *H. influenzae* meningitis instead of streptococcal infection, and the ages and countries of origin for patients with macroprolactinomas presenting initially as bacterial meningitis make it likely that most were unvaccinated against *H. influenzae*.

CSF rhinorrhea occurred in 5/14 (36%) of macroprolactinomas initially presenting as bacterial meningitis, substantially higher than the rate of spontaneous CSF rhinorrhea (2.6%) reported in a large series of invasive macroprolactinomas uncomplicated by meningitis [19]. However, our patient experienced CSF rhinorrhea after starting treatment with the potent dopamine agonist cabergoline, and this complication occurred in slightly more than 5% of invasive macroprolactinomas initially treated with dopamine agonists [19]. Due to the risk of tumor regression and CSF fistula, it is reasonable to consider surgical management of an invasive prolactinoma before starting dopamine agonist treatment [7].

The mortality rate among patients with macroprolactinomas presenting as bacterial meningitis is 14% (2/14). There are too few deaths in this series to determine clinical factors that might predispose to mortality in these cases. Also, there is little published in the peer-reviewed literature regarding the mortality rate for patients with bacterial meningitis due to occult pituitary adenomas. In a recent case series of patients with pituitary macroadenomas presenting with bacterial meningitis, including some patients with macroprolactinomas, the mortality rate was 12.5% (2/16) [20].

## Conclusions

Though invasive macroprolactinomas are well described in MEN1, this appears to be the first report of MEN1 diagnosed in the setting of bacterial meningitis as the initial presentation of a macroprolactinoma. This is also the first case of macroprolactinoma presenting with bacterial meningitis in which *H. influenzae* was the etiology instead of a streptococcal species. The patient's course illustrates that surgery to resect as much tumor as possible and repair bony defects should be considered before starting dopamine agonist treatment due to the potential for a CSF fistula to occur. MEN1 should be considered in any case of a pituitary mass occurring with hypercalcemia.
